# Inhibition of HMGB1 protects the retina from ischemia-reperfusion, as well as reduces insulin resistance proteins

**DOI:** 10.1371/journal.pone.0178236

**Published:** 2017-05-23

**Authors:** Li Liu, Youde Jiang, Jena J. Steinle

**Affiliations:** 1 Department of Anatomy and Cell Biology, Wayne State University School of Medicine, Detroit, Michigan, United States of America; 2 Department of Ophthalmology Wayne State University School of Medicine, Detroit, Michigan, United States of America; Virgen Macarena University Hospital, School of Medicine, University of Seville, SPAIN

## Abstract

The role of inflammation in diabetic retinal amage is well accepted. While a number of cytokines and inflammatory mediators are responsible for these changes, upstream regulators are less well studied. Additionally, the role for these upstream mediators in retinal health is unclear. In this study, we hypothesized that inhibition of high mobility group box 1 (HMGB1) could restore normal insulin signaling in retinal endothelial cells (REC) grown in high glucose, as well as protect the retina against ischemia/reperfusion (I/R)-induced retinal damage. REC were grown in normal (5mM) or high glucose (25mM) and treated with Box A or glycyrrhizin, two different HMGB1 inhibitors. Western blotting was done for HMGB1, toll-like receptor 4 (TLR4), insulin receptor, insulin receptor substrate-1 (IRS-1), and Akt. ELISA analyses were done for tumor necrosis factor alpha (TNFα) and cleaved caspase 3. In addition, C57/B6 mice were treated with glycyrrhizin, both before and after ocular I/R. Two days following I/R, retinal sections were processed for neuronal changes, while vascular damage was measured at 10 days post-I/R. Results demonstrate that both Box A and glycyrrhizin reduced HMGB1, TLR4, and TNFα levels in REC grown in high glucose. This led to reduced cleavage of caspase 3 and IRS-1^Ser307^ phosphorylation, and increased insulin receptor and Akt phosphorylation. Glycyrrhizin treatment significantly reduced loss of retinal thickness and degenerate capillary numbers in mice exposed to I/R. Taken together, these results suggest that inhibition of HMGB1 can reduce retinal insulin resistance, as well as protect the retina against I/R-induced damage.

## Introduction

The role of inflammation as a key factor in diabetic retinopathy has become of increasing importance [1, 2]. While it is clear that a number of proteins, including TNFα and IL1β, are involved in the pathogenesis of diabetic retinopathy, the upstream regulators of these inflammatory mediators are less clear. Additionally, a role of innate immunity in the retinal damage and insulin resistance has come into focus [3–6]. Additionally, it is clear that the increased TNFα noted in the diabetic retina can lead to impaired insulin signaling in the retina through phosphorylation of IRS-1 on serine 307 in retinal endothelial cells (REC) [7].

One potential upstream regulator of TNFα and insulin resistance is high mobility group box 1 (HMGB1) [6, 8, 9]. Work has shown that C57/BL6 mice treated with anti-HMGB1 and fed a high fat diet had decreased hepatic TNFα and MCP-1 levels, despite the high fat diet, suggesting that HMGB1 can drive TNFα and liver inflammation [8]. Work in cultured adipocytes from humans showed that lean humans has increased levels of nuclear HMGB1 vs. obese individuals, who had predominately cytosolic HMGB1 [9]. Increased cytosolic HMGB1 is associated with increased inflammation.

Since HMGB1 is associated with increased inflammation, a number of agents have been developed to inhibit HMGB1 actions. The Box A portion of HMGB1 competes with full length HMGB1 for binding sites, demonstrating that Box A serves as an anti-inflammatory agent [6]. Work in the ischemic/reperfusion (I/R) model of heart disease showed that Box A treatment protected the heart, likely through reduced c-Jun N-terminal kinase (JNK) [10]. Similarly, Box A treatment in a model of middle cerebral artery occlusion demonstrated that inhibition of HMGB1 with Box A protected the ischemic brain [11]. In addition to Box A, glycyrrhizin has been suggested as a HMGB1 inhibitor. Glycyrrhizin is a natural triterpene found in roots and rhizones of licorice. It inhibits HMGB1 by binding directly to both HMG boxes [12]. Work in 1-month diabetic rats showed that glycyrrhizin significantly reduced HMGB1, ERK1/2, cleaved caspase 3, and glutamate levels [13]. Additionally, work in receptor for advanced glycation end products (RAGE) knockout mice showed that I/R caused a significant increase in HMGB1 levels in the retina, which was attenuated by a HMGB1 neutralizing antibody [14]. Inhibition of HMGB1 also reduced neuronal cell loss in the mice.

To test whether HMGB1 plays a role in insulin resistance and retinal damage, we treated REC cultured in high glucose with Box A or glycyrrhizin and measured key insulin signaling proteins. Additionally, we used the I/R model of retinal damage with glycyrrhizin treatment to investigate whether HMGB1 inhibition could reduce neuronal and vascular damage to the retina.

## Methods

### Retinal endothelial cell culture

Primary human retinal endothelial cells (REC) were acquired from Cell Systems Corporation (CSC, Kirkland, Washington). Cells were grown in Cell Systems medium supplemented with microvascular growth factors (MVGS), 10ug/mL gentamycin, and 0.25ug/mL amphotericin B (Invitrogen, Carlsbad, CA). Once cells reached confluence, some dishes were moved to Cell Systems Medium with supplemented D-glucose to 25mM. Only dishes prior to passage 6 were used. Cells were quiesced by incubating in high or normal glucose medium without MVGS for 24 h prior to experimental use.

### Cell culture treatments

REC in normal (5mM) and high glucose (25mM) treated with Box A (10nM for 2 hours)[15] or glycyrrhizin (2mM for 2 hours)[16].

### ELISA

A TNFα ELISA (Fisher Scientific, Pittsburgh, PA) was used according to manufacturer’s instructions on cell lysates (collected into RIPA lysis buffer) with the exception that sample exposure to primary antibody occurred for 24hrs. One hundred micrograms of protein were used to insure equal protein amounts in all wells. A cleaved caspase 3 ELISA (Cell Signaling Technology, Danvers, MA) was done according to manufacturer’s instructions with equal protein concentrations loaded in all wells so analyses could be done using the optical density (O.D.)

### Western blotting

After Box A or glycyrrhizin treatment and rinsing with cold phosphate-buffered saline, REC were collected into lysis buffer (RIPA buffer) containing protease and phosphatase inhibitors and frozen at -20C. For experiments, equal amounts of protein from the cell extracts were separated on the pre-cast tris-glycine gel (Invitrogen, Carlsbad, CA), blotted onto a nitrocellulose membrane. After blocking in TBST (10mM Tris-HCl buffer, pH 8.0, 150 mM NaCl, 0.1% Tween 20) and 5% (w/v) BSA, the membranes was treated with total insulin receptor, phosphorylated insulin receptor (Tyr 1150/1151), total Akt, phosphorylated Akt (Ser473, all from Cell Signaling, Danvers, MA)), HMGB1, and TLR4 (from Abcam, Cambridge, MA) primary antibodies followed by incubation with secondary antibodies labeled with horseradish peroxidase. Antigen-antibody complexes were detected by chemilluminescence reagent kit (Thermo Scientific, Pittsburgh, PA). Data was acquired using an Azure C500 (Azure Biosystems, Dublin, CA).

### Mice

All animal procedures meet the Association for Research in Vision and Ophthalmology requirements and were approved by the Institutional Animal Care and Use Committee of Wayne State University (A-08-07-15) and conform to NIH guidelines. Twenty male C57/B6 mice were purchased from Charles River at 8 weeks of age. Mice were allowed to acclimate to the vivarium at Wayne State University for 1 week prior to initiation of treatments. Euthanasia of all mice was done by CO_2_, followed by cervical dislocation.

### Ischemia/Reperfusion model (I/R)

Animals were anesthetized with an intraperitoneal injection of ketamine (90mg/kg) and xylazine (8mg/kg). Once animals did not have a toe pinch reflex, the anterior chamber of the eye was cannulated with a 32-gauge needle attached to an infusion line of sterile saline. The eye was subjected to 90 minutes of hydrostatic pressure (80–90mmHg, TonoPen, Medtronic, Jacksonville, FL) to induce retinal ischemia as evidenced by blanching of the iris and loss of red reflex [17, 18]. After 90 minutes, the needle was withdrawn and intraocular pressure normalized, causing reperfusion. The contralateral eye served as an in-animal control (labeled as control).

### Treatments to mice

10mg/kg glycyrrhizin dissolved in PBS was given I.P. 2 hours prior to the initiation of I/R and once a day for two days following I/R to 10 mice. Data is shown for the contralateral eye (ctrl+Gly) and the eye exposed to I/R after glycyrrhizin (IR+Gly).

### Neuronal analyses

Two days after I/R exposure, a subset of each group of mice was sacrificed for measurements of neuronal thickness, as we have done previously [19]. Briefly, the whole eye is removed and placed into formalin. The retina is separated from the rest of the globe and embedded in O.C.T. freezing medium. Ten micrometer sections were taken from throughout the retina, followed by hematoxylin and eosin staining. Analyses were done on 10 sections from 5 animals in each group as we have done in the past [18, 19].

### Vascular analyses

Ten days after I/R exposure, the remaining mice (5 from each group) were sacrificed to measure degenerate capillaries, as we have done previously. Briefly, degenerate capillaries were counted in mid-retina in six to seven fields evenly spaced around the retina. Degenerate capillaries were identified as capillary-sized tubes having no nuclei anywhere along their length. Degenerate capillaries were counted only if their average diameter was at least 20% of that found in surrounding healthy capillaries. [18, 20].

### Statistical analyses

Non-parametric Kruskal-Wallis with Dunn’s post-hoc tests were used for the cell culture data. One-way ANOVA with a Student Newman Keul’s post-hoc test was done for animal studies. P<0.05 was considered statistically significant.

## Results

### Both Box A and glycyrrhizin significantly reduced HMGB1 and TLR4 levels

**Fig 1:**
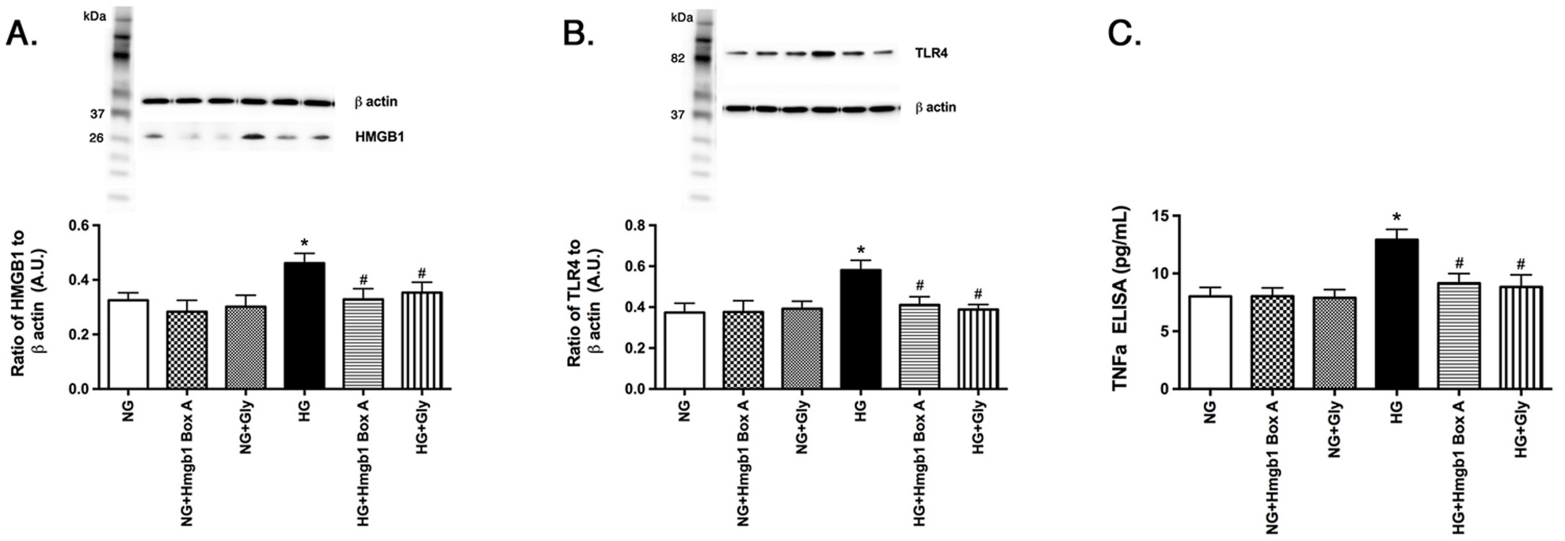
Box A and glycyrrhzin can reduce HMGB1, TLR4 and TNFα in REC grown in high glucose. REC were grown in normal (NG) or high glucose (HG) and treated with Box A (Box A) or glycyrhizzin (Gly). Western blotting was done for HMGB1 (A) and TLR4 (B) levels. ELISA analyses were done on REC for TNFα levels. Data are mean ± SEM. *P<0.05 vs NG, #P<0.05 vs. HG. N = 4 for all groups.

Literature suggested that both Box A and glycyrrhizin inhibits HMGB1 in other targets [10, 14]. We wanted to investigate whether these agents could reduce HMGB1 in REC cultured in high glucose. Fig 1A shows high glucose significantly increased HMGB1 levels in REC, which was inhibited by both Box A and glycyrrhizin. Both Box A and glycyrrhizin also were able to significantly reduce the high glucose-induced increase in TLR4 (Fig 1B). Since activation of both HMGB1 and TLR4 often activates inflammatory pathways [21], we also show that both Box A and glycyrrhizin inhibited TNFα levels in REC grown in high glucose (P<0.05 vs. HG) (Fig 1C).

### Inhibition of HMGB1 restored normal insulin receptor phosphorylation and reduced IRS-1^Ser307^ phosphorylation

**Fig 2:**
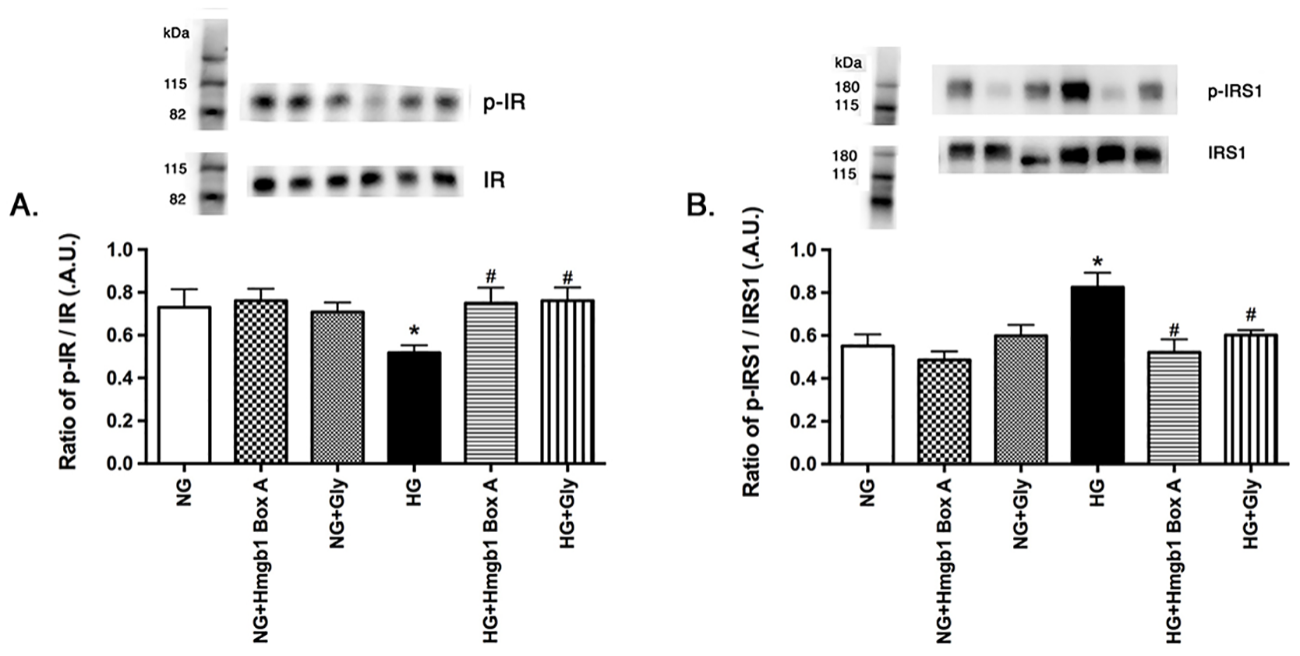
HMGB1 inhibition restored insulin receptor phosphorylation, while reducing IRS-1^Ser307^ in REC. Panel A is Western blot results for insulin receptor, while panel B shows Western blot results for IRS-1^Ser307^. Data are mean ± SEM. *P<0.05 vs NG, #P<0.05 vs. HG. N = 4 for all groups.

We have previously reported that high glucose reduced insulin receptor phosphorylation, as well as increased IRS-1^Ser307^ activation [7] in REC. Others have also suggested that HMGB1 may be involved in impaired insulin signaling [6]. Thus, we investigated whether Box A and glycyrrhizin could inhibit the high glucose-induced loss of insulin receptor activation. Fig 2A shows that inhibition of HMGB1 by either agent significantly increased insulin receptor phosphorylation in REC cultured in high glucose (P<0.05 vs. HG). Potentially through the reduction in TNFα levels, both Box A and glycyrrhizin significantly reduced IRS-1^Ser307^ phosphorylation, suggesting that both agents can improve insulin receptor signaling in REC.

### Akt phosphorylation is restored with reduced cleaved caspase 3 observed after HMGB1 inhibition

**Fig 3:**
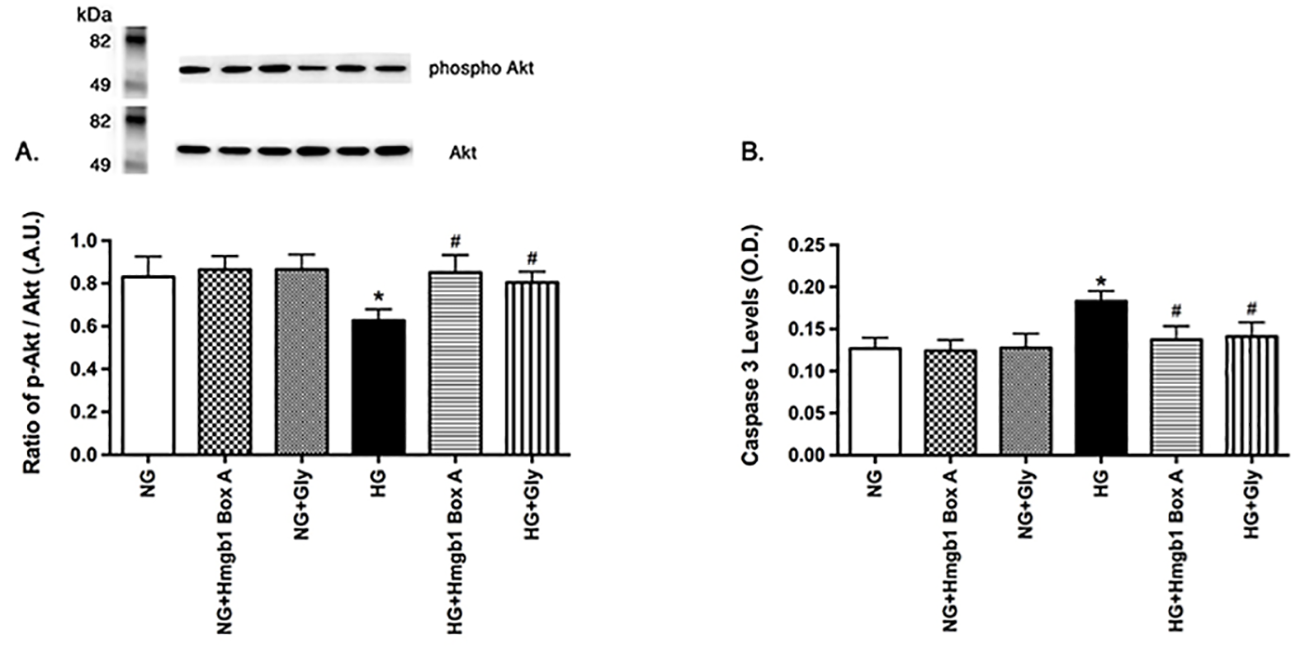
Inhibition of HMGB1 by Box A and glycyrrhizin significantly reduced cleaved caspase 3 levels, likely due to the increased Akt phosphorylation in REC. Panel A is Western blot results for Akt phosphorylation, while panel B shows ELISA results for cleaved caspase 3. Data are mean ± SEM. *P<0.05 vs NG, #P<0.05 vs. HG. N = 4 for all groups.

With the restoration of insulin receptor activation following HMGB1 inhibition, it was key to determine if downstream factors were also affected. Fig 3A shows that both Box A and glycyrrhizin blocked the high glucose-induced loss of Akt phosphorylation (P<0.05 vs. HG). The increase in Akt phosphorylation after HMGB1 inhibition led to a significant decrease in the cleavage of caspase 3 (P<0.05 vs. HG, Fig 3B).

### Glycyrrhizin protected retina against ischemia/reperfusion-induced loss of retinal thickness and cell numbers in the ganglion cell layer

**Fig 4:**
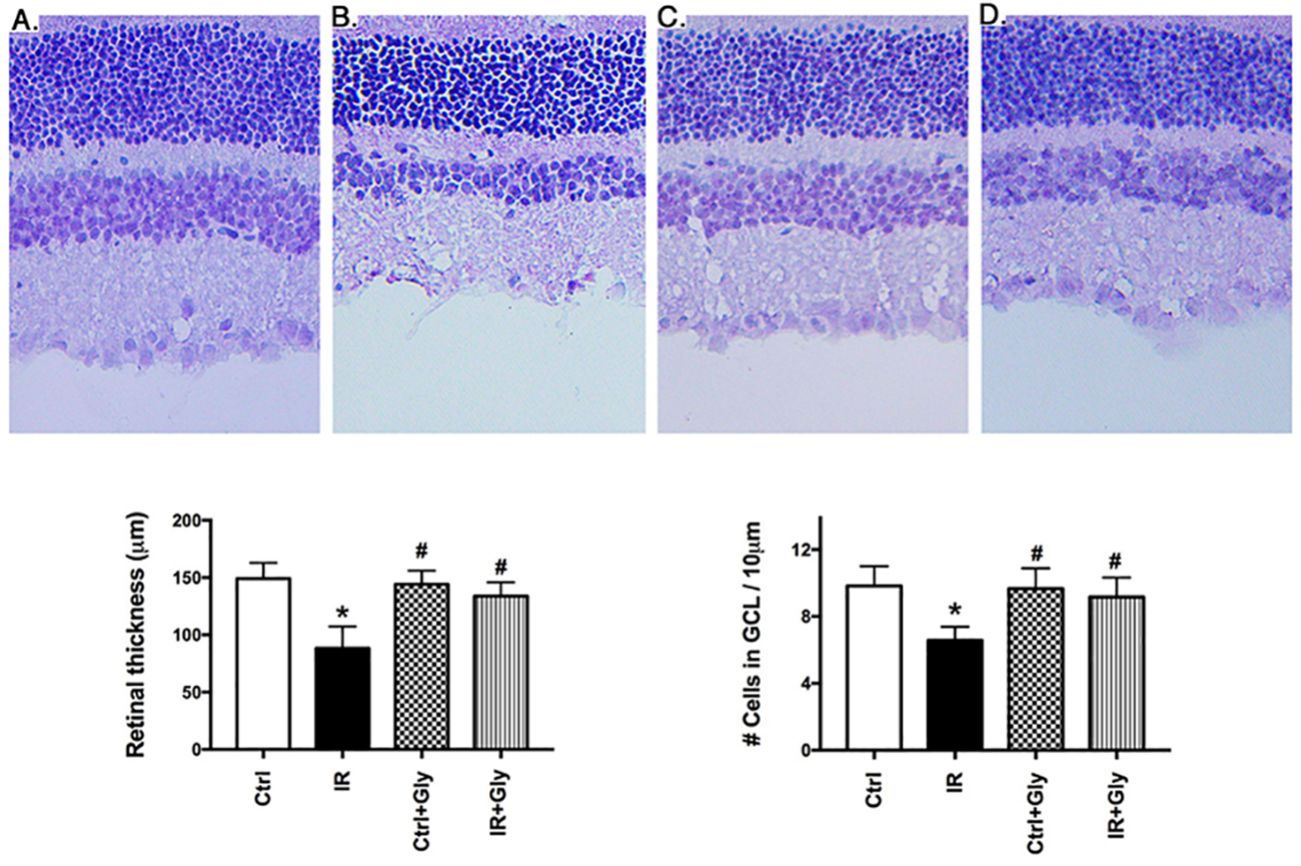
Glycyrrhizin treatment restored retinal thickness and cell numbers in the ganglion cell layer in C57BL/6J mice after ischemia/reperfusion treatment. Image A shows the contralateral eye (ctrl), while B demonstrated the effects of I/R on retinal thickness (IR). Panel C is the contralateral eye after glycyrrhizin treatment (ctrl+Gly). Panel D shows an image from mice treated with glycyrrhizin before I/R (IR+Gly). Bar graphs below quantitate changes in retinal thickness (left) and cell numbers (right). Data are mean ± SEM. *P<0.05 vs ctrl, #P<0.05 vs. IR. N = 5 for all groups.

Exposure to ischemia/reperfusion significantly decreases retinal thickness and numbers of cells in the ganglion cell layer [18, 22]. Since we found that glycyrrhizin inhibited HMGB1, TLR4, and TNFα in REC cultured in high glucose, we wanted to test whether glycyrrhizin would protect the retina from I/R injury. Fig 4A and 4B show that I/R caused significant thinning of the retina. Glycyrrhizin treatment blocked the loss of retinal thickness (Fig 4C), thus protecting the retina. Additionally, glycyrrhizin was able to prevent the loss of cell numbers in the ganglion cell layer due to I/R (P<0.05 vs. I/R only). Glycyrrhizin treatment did not affect the contralateral retina.

### Retinal vascular damage following I/R is reduced following treatment with glycyrrhizin

**Fig 5:**
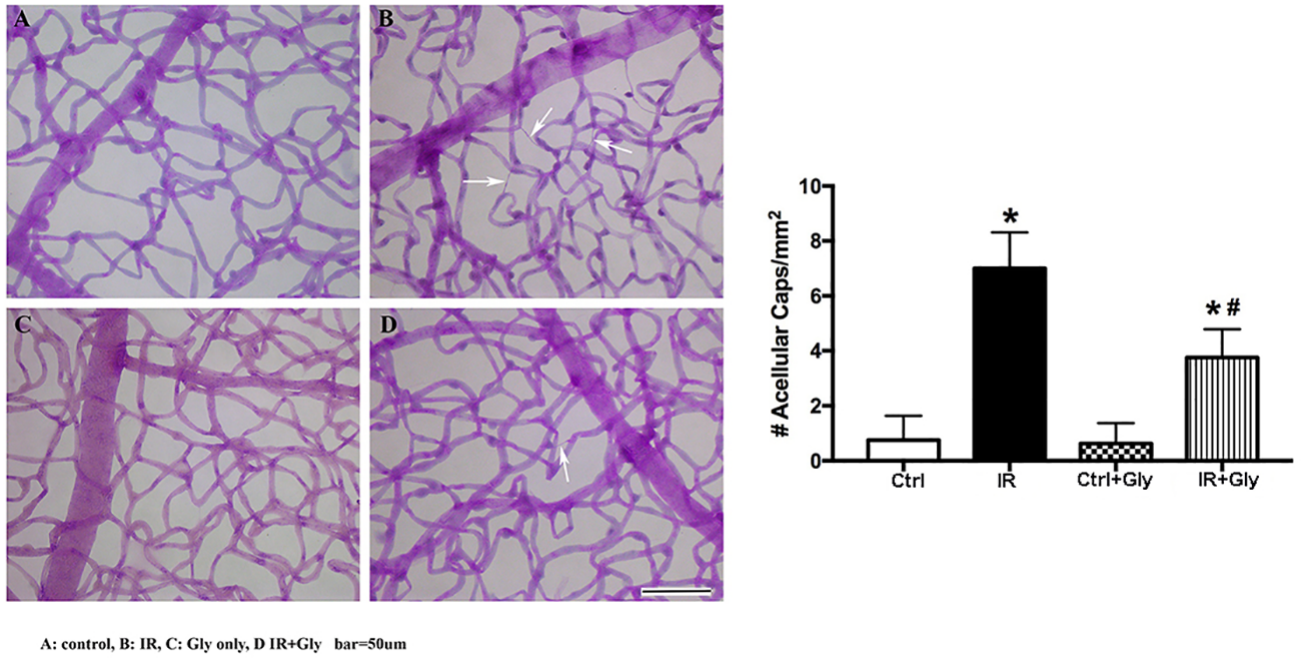
Glycyrrhizin protects the retinal vasculature of C57BL/6J mice. Panels A and B show the effects of I/R on the retinal vasculature. (A) is the contralateral eye (ctrl) and (B) is the eye exposed to I/R (IR). Panel C is the contralateral eye after glycyrrhizin treatment (ctrl+Gly). Panel D shows an image from mice treated with glycyrrhizin before I/R (IR+Gly). Quantitation of the numbers of degenerate capillaries is presented in the bar graphs. Data are mean ± SEM. *P<0.05 vs ctrl (control), #P<0.05 vs. IR. N = 5 for all groups.

As glycyrrhizin protected REC, we also investigated the role of glycyrrhizin on the retinal vasculature *in vivo*. Fig 5C vs. 5B shows that glycyrrhizin treatment significantly reduced (P<0.05 vs. I/R) the numbers of degenerate capillaries compared to retina exposed to I/R, suggesting that inhibition of HMGB1 is protective to a damaged retina.

## Discussion

The role of chronic inflammation in diabetic retinopathy is well established [1, 2]. While the retinal damage of diabetes can be ameliorated through use of anti-inflammatory agents in rodents [17, 23], many of these agents are not amenable therapy development since these same inflammatory factors are required to protect the body in times of stress. Thus, work has focused on the cellular mechanisms by which these inflammatory mediators cause retinal damage. One of the goals of this study was to investigate whether inhibition of HMGB1 could restore normal insulin signaling. We have previously demonstrated that TNFα leads to IRS-1^Ser307^ phosphorylation, leading to decreased Akt activation and apoptosis of REC [7]. This response could be inhibited by pioglitazone [24] or salicylate [25] in the retina from type 2 diabetic rats. While TNFα can be regulated by a large number of factors, we recently demonstrated that Compound 49b, a β-adrenergic receptor agonist, reduced both TLR4 and TNFα levels [3]. Since HMGB1 often lies upstream of both TLR4 and TNFα [26], we hypothesized that inhibition of HMGB1 may reduce TNFα levels, leading to increased insulin receptor phosphorylation and reduced REC apoptosis. Indeed, we found that use of Box A or glycyrrhizin could significantly reduce HMGB1, TLR4, and TNFα levels. These same HMGB1 antagonists significantly reduced IRS-1^Ser307^ phosphorylation, leading to reduced cleavage of caspase 3 in REC grown in high glucose. Our findings in REC agree with work in liver inflammation, where anti-HMGB1 antibody treatment to mice on a high fat diet led to reduced hepatic TNFα and MCP-1 levels [8]. Similarly, work in SW872 fat cells showed that HMGB1 can serve as an adipokine [27], which often cause insulin resistance. However, in the SW872 cell line, IL-6 was responsible for the inflammation, rather than TLR2 or TLR4. Work in diabetic rats injected with HMGB1 showed increased cleavage of caspase 3; however, insulin receptor actions were not studied in that study [13]. Thus, while the actions of HMGB1 on insulin resistance remain understudied, work from multiple systems suggests that HMGB1 can regulate TNFα and that HMGB1 can be linked to members of the insulin resistance pathway.

In addition to the focus on insulin signaling, this study also demonstrated that glycyrrhizin treatment protected against I/R-induced retinal damage of mice. These findings agree with work in RAGE knockout mice [14], where HMGB1 caused damage to retinal ganglion cells in the I/R model, which was attenuated in RAGE knockout mice. Work in a mouse model of cerebral ischemia showed that Box A was effective in reducing ischemic brain damage through a reduction in RAGE activity [11]. Work in the heart also demonstrated a protective role for HGMB1 inhibition against I/R-induced damage. Thus, the findings of reduced neuronal and vascular retinal damage in mice treated with glycyrrhizin follow work in other models. We have also previously published that Compound 49b, a novel β-adrenergic receptor agonist, protected the retina from I/R-induced damage [18]. We have shown that Compound 49b can restore normal insulin signaling in vivo [28]. We have also reported that a β1-adrenergic receptor agonist, xamoterol, could regulate insulin signaling in REC grown in high glucose. Taken together, β-adrenergic receptors may lie upstream of HMGB1, which would explain why both Compound 49b and glycyrrhizin protect the retina against I/R-induced damage.

This study has a few limitations. Since we did both a pre- and post-treatment with glycyrrhizin to insure we had sufficient HMGB1 inhibition, future studies will focus on only pre-treatment vs. post-treatment effects of glycyrrhizin to allow for better therapeutic development. Additionally, since the entire retina is needed for the neuronal and vascular analyses, we could not measure insulin resistance proteins after I/R. We will pursue these measurements from diabetic mice treated with glycyrrhizin in the future.

In conclusion, the results of the present study were two-fold. Data demonstrate that inhibition of HMGB1 reduced inflammatory mediators, leading to increased insulin receptor signal transduction in REC grown in high glucose. Secondly, use of glycyrrhizin was an effective therapy against I/R-induced retinal neuronal and vascular damage. Taken together, these findings suggest that additional work on HMGB1 and anti-HMGB1 agents may lead to novel therapies for some of the retinal complications of diabetic retinopathy.

## Abbreviations

GCL: ganglion cell layer
HMGB1: high mobility group box 1
I/R: ischemia/reperfusion
IR: insulin receptor
IRS-1: insulin receptor substrate-1
REC: retinal endothelial cell
TLR4: toll-like receptor 4
TNFα: tumor necrosis factor alpha
